# An Account of the Arrangement of the Muscular Substance in the Urinary and Certain of the Generative Organs of the Human Body

**Published:** 1856-09

**Authors:** George Viner Ellis

**Affiliations:** Professor of Anatomy in University College, London


*An Account of the Arrangement of the Muscular Substance in the Urinary and Certain of the Generative Organs of the Human Body.* By George Viner Ellis, Esq., F. R. C. S., Professor of Anatomy in University College, London.

It would be vain to attempt to represent this important communication by an abstract; all that can be done is to draw attention to some of the most striking points in it. After a minute description of the three more or less perfect strata of involuntary muscular fibres which constitute the muscular substance of the bladder—viz., an external or longitudinal, a middle or circular, and an internal longitudinal or submucous, stratum—the author proceeds to trace them through the rest of the genito-urinary apparatus. With respect to the prostate, after a minute description of its structure, the author deduces that it is less of a glandular than of a muscular body, and is only a largely developed portion of the circular muscular layer that invests all the urethra behind the bulb or spongy portion, and which is continuous, without interruption, with the circular fibres of the bladder. As the prostatic enlargement includes only part of that muscular stratum of the urethra, the author proposes the name *orbicularis, vel sphincter urethræ,* for both the prostate and the prolongation around the membranous portion of the urethra; while he would confine the old term *prostate* (without the word *gland*) to the thickened and more powerful part near the neck of the bladder. The submucous layer of the bladder is traced throughout the whole length of the urethra. A muscular covering of the vesiculæ and vasa deferentia, consisting of two layers of fibres, (one longitudinal, the other transverse,) is next described; and the paper concludes with a very elaborate description of the sheaths surrounding the spongy structure of the penis.

Mr. Quain was very glad that Mr. Ellis, of whose accuracy the Profession might be assured, had given so good a description (which had long been wanted) of the muscular structure of the prostate gland, and he should be glad to see, from the same pen, or from some other, an account of the glandular structure.—*London Med. Times and Gazette.*

